# Association of Mean Corpuscular Hemoglobin Concentration With Mortality in Patients With Cancer

**DOI:** 10.7759/cureus.104050

**Published:** 2026-02-22

**Authors:** Jing Zhang, Shaohua Zhang, Sheng Chen, Zhuchun Jiang

**Affiliations:** 1 Graduate School, Guangxi University of Chinese Medicine, Nanning, CHN; 2 Oncology Department, Ruikang Hospital, Guangxi University of Chinese Medicine, Nanning, CHN

**Keywords:** cancer, eicu-crd, icu, mchc, mean corpuscular hemoglobin concentration, mortality, retrospective cohort study

## Abstract

Background: While mean corpuscular hemoglobin concentration (MCHC) correlates with prognosis in various critical conditions, its relationship with mortality in ICU patients with cancer remains underexplored.

Methods: We included 20,055 cancer patients with available MCHC values. Data extracted included demographics, comorbidities, laboratory parameters within 24 hours, illness severity scores, and vasoactive medication use. Primary outcomes were 30-day and 90-day in-hospital mortality. Kaplan-Meier analysis and multivariable Cox proportional hazards models assessed the association between MCHC and mortality.

Results: Kaplan-Meier curves showed significant survival differences across MCHC quartiles, with higher MCHC associated with better survival. In fully adjusted Cox models, each unit increase in MCHC was associated with lower mortality risk at 30 days (HR: 0.86, 95% CI: 0.83-0.89) and 90 days (HR: 0.86, 95% CI: 0.83-0.88). Patients in the highest MCHC quartile had significantly lower mortality risk compared to the lowest quartile (30-day - HR: 0.56, 95% CI: 0.49-0.63; 90-day - HR: 0.55, 95% CI: 0.49-0.62). Subgroup analyses confirmed consistent associations across all clinical strata.

Conclusion: Lower MCHC levels at ICU admission are independently associated with increased short-term and medium-term mortality in critically ill cancer patients.

## Introduction

Recent advances in anti-cancer therapeutics, together with substantial improvements in supportive care strategies, have significantly enhanced overall survival rates among patients with cancer [1]. Critically ill cancer patients can still derive meaningful short-term benefits from ICU care that are comparable to those observed in non-cancer patients. Moreover, evidence suggests that, among critically ill individuals, the short-term outcomes of patients with solid tumors admitted to the ICU are similar to those of ICU patients admitted for other non-oncological conditions, indicating that ICU interventions can be equally effective in this subgroup [2,3].

Mean corpuscular hemoglobin concentration (MCHC), which is widely used as a routine hematological parameter and serves as a common indicator for the evaluation of anemia, has been increasingly recognized for its prognostic relevance in a variety of clinical conditions. Previous studies have demonstrated that MCHC is significantly associated with clinical outcomes and prognosis in patients with acute myocardial infarction [4], acute pulmonary embolism [5], hypertension [6], and hepatorenal syndrome [7], suggesting that this readily available laboratory index may reflect underlying pathophysiological changes that influence disease severity and patient outcomes.

A focused investigation into the association between MCHC and mortality in this vulnerable population remains absent from the current literature.

## Materials and methods

Data source

The eICU Collaborative Research Database (eICU-CRD) offers a substantial volume of high-quality clinical data and provides researchers with detailed and structured information, making it particularly suitable for in-depth analyses of ICU populations and specific disease conditions, including patients with cancer [8].

Data collection was done using authorized access to the eICU-CRD, for which author Sheng Chen completed the mandatory human subjects protection training (ID: 66963781) and secured credentialed user status on PhysioNet.

The eICU-CRD database contains extensive and detailed patient-level information, including but not limited to hospital length of stay, results of a wide range of laboratory examinations, records of medication administration, and continuous or intermittent measurements of vital signs.

Participants

Any recorded malignancy based on the International Classification of Diseases, Ninth Revision, Clinical Modification (ICD-9-CM) and International Classification of Diseases, Tenth Revision, Clinical Modification (ICD-10-CM) diagnostic codes was included. The study excluded the following patient groups: (1) minors (age < 18 years), because pediatric patients often differ substantially from adults in terms of disease characteristics, physiological responses, and treatment strategies, and their inclusion could introduce confounding factors that might affect the interpretability and generalizability of the findings; (2) patients with multiple ICU admissions; only the first admission was analyzed to avoid bias from subsequent stays obscuring the relationship between baseline MCHC and mortality; and (3) patients without a documented MCHC measurement in their medical records. Excluding patients lacking MCHC data ensured that all individuals included in the analysis had complete and relevant laboratory information, thereby allowing for a consistent and accurate assessment of the relationship under investigation. Collectively, these carefully defined inclusion and exclusion criteria were implemented to strengthen the methodological rigor of the study and to improve the clarity and robustness of the resulting conclusions.

Missing data

Multiple imputation techniques were applied to address missing data for variables in which the proportion of missing values was less than 20%, thereby reducing potential bias and improving the robustness and reliability of the statistical analyses [9].

Clinical outcomes

This study defined 30-day and 90-day in-hospital mortality as its primary endpoints, with the objective of evaluating short- and intermediate-term survival outcomes among the enrolled ICU patients with cancer. These time-specific mortality measures allowed for a more detailed assessment of early prognosis and facilitated the examination of mortality risk across different clinically relevant follow-up periods.

The secondary endpoint of the study was all-cause mortality during hospitalization, which was included to provide a comprehensive evaluation of overall in-hospital survival regardless of the specific cause of death. This endpoint enabled a broader assessment of patient outcomes and complemented the primary endpoints by capturing the full spectrum of mortality events occurring during the hospital stay.

## Results

Characteristics of the patients

**Figure 1:**
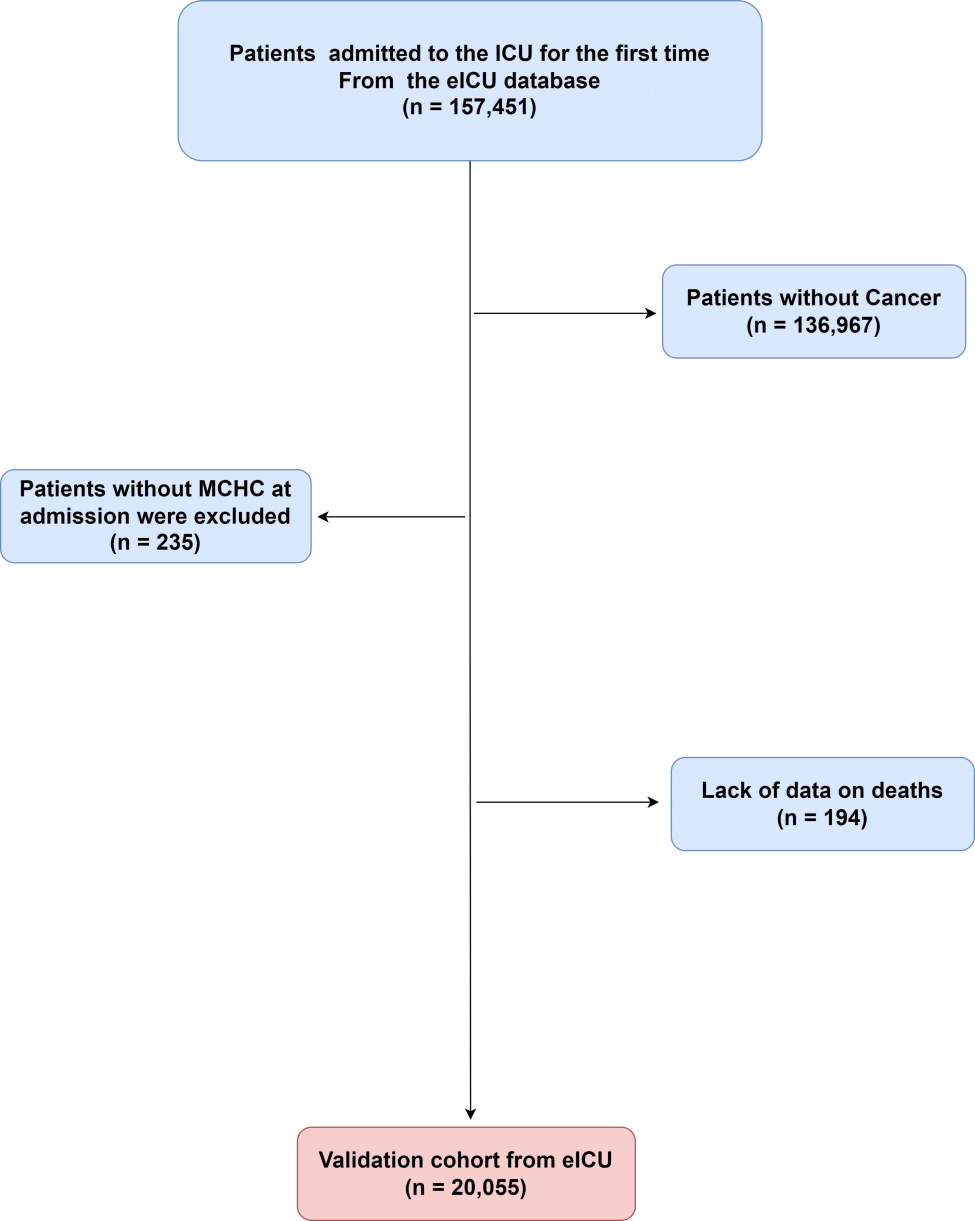
Study population enrollment flowchart. ICU: intensive care unit; MCHC: mean corpuscular hemoglobin concentration.

A total of 20,055 patients from the initial dataset met all criteria and were included in the final analysis, as shown in Figure 1.

The overall in-hospital mortality rate was 13.15% (2,639/20,055), indicative of a critically ill population. Baseline characteristics are shown in Table 1.

**Table 1:** Baseline characteristics of survivors and deceased patients. Note: "-" indicates empty cells. Data are presented as standard deviation (SD) or frequencies (percentages). MCHC: mean corpuscular hemoglobin concentration; SOFA: Sequential Organ Failure Assessment; CCI: Charlson Comorbidity Index; SAPS II: Simplified Acute Physiology Score II; OASIS: Oxford Acute Severity of Illness Score; WBC: white blood cells; RBC: red blood cells; AF: atrial fibrillation; RF: respiratory failure; HF: heart failure; DM: diabetes mellitus.

Variable	Total (n = 20055)	Alive (n = 17416)	Death (n = 2639)	p-value
MCHC	33.13 ± 1.66	33.17 ± 1.34	32.90 ± 3.01	<0.0001
Sex				0.04
Female	9496 (47.35)	8297 (47.64)	1199 (45.43)	-
Male	10559 (52.65)	9119 (52.36)	1440 (54.57)	-
Age	69.64 ± 12.99	69.44 ± 13.02	70.95 ± 12.71	<0.0001
Weight	79.68 ± 23.62	80.02 ± 23.69	77.46 ± 23.00	<0.0001
Comorbidities				
RF				<0.0001
No	15600 (77.79)	14157 (81.29)	1443 (54.68)	-
Yes	4455 (22.21)	3259 (18.71)	1196 (45.32)	-
HF				<0.0001
No	18529 (92.39)	16147 (92.71)	2382 (90.26)	-
Yes	1526 (7.61)	1269 (7.29)	257 (9.74)	-
AF				<0.0001
No	17961 (89.56)	15683 (90.05)	2278 (86.32)	-
Yes	2094 (10.44)	1733 (9.95)	361 (13.68)	-
DM				0.69
No	18208 (90.79)	15818 (90.82)	2390 (90.56)	-
Yes	1847 (9.21)	1598 (9.18)	249 (9.44)	-
Paraplegia				0.54
No	20037 (99.91)	17399 (99.90)	2638 (99.96)	-
Yes	18 (0.09)	17 (0.10)	1 (0.04)	-
Sepsis				<0.0001
No	16758 (83.56)	14872 (85.39)	1886 (71.47)	-
Yes	3297 (16.44)	2544 (14.61)	753 (28.53)	-
Stroke				<0.0001
No	19172 (95.60)	16704 (95.91)	2468 (93.52)	-
Yes	883 (4.40)	712 (4.09)	171 (6.48)	-
Laboratory tests				
SAPS II	32.00 (24.00, 42.00)	31.00 (23.00, 39.00)	48.00 (36.00, 61.00)	<0.0001
SOFA	5.00 (3.00, 7.00)	5.00 (3.00, 7.00)	8.00 (5.00, 11.00)	<0.0001
CCI	7.00 (5.00, 9.00)	6.00 (5.00, 9.00)	8.00 (6.00, 10.00)	<0.0001
OASIS	25.00 (19.00, 32.00)	24.00 (19.00, 30.00)	33.00 (26.00, 42.00)	<0.0001
WBC	11.30 (8.00, 15.50)	11.10 (7.97, 15.10)	13.39 (8.60, 19.00)	<0.0001
RBC	3.74 ± 0.72	3.76 ± 0.71	3.62 ± 0.75	<0.0001
Hemoglobin	11.23 ± 2.09	11.29 ± 2.08	10.79 ± 2.15	<0.0001
Platelet	204.00 (151.00, 266.05)	205.00 (154.00, 265.05)	196.80 (123.00, 274.00)	<0.0001
Glucose	156.00 (125.00, 201.00)	155.00 (124.00, 197.00)	167.00 (130.00, 224.00)	<0.0001
Sodium	138.85 ± 4.91	138.79 ± 4.66	139.22 ± 6.29	<0.001
Creatinine	1.10 (0.80, 1.65)	1.05 (0.78, 1.54)	1.50 (0.97, 2.37)	<0.0001
Drug use				
Vasopressin				<0.0001
No	19173 (95.60)	17017 (97.71)	2156 (81.70)	-
Yes	882 (4.40)	399 (2.29)	483 (18.30)	-
Dopamine				<0.0001
No	19193 (95.70)	16783 (96.37)	2410 (91.32)	-
Yes	862 (4.30)	633 (3.63)	229 (8.68)	-
Epinephrine				<0.0001
No	19212 (95.80)	16839 (96.69)	2373 (89.92)	-
Yes	843 (4.20)	577 (3.31)	266 (10.08)	-

The presentation of these baseline data enhances the transparency of the analysis and enables readers to evaluate the potential impact of baseline differences on clinical outcomes.

Kaplan-Meier survival analysis curves for mortality

**Figure 2:**
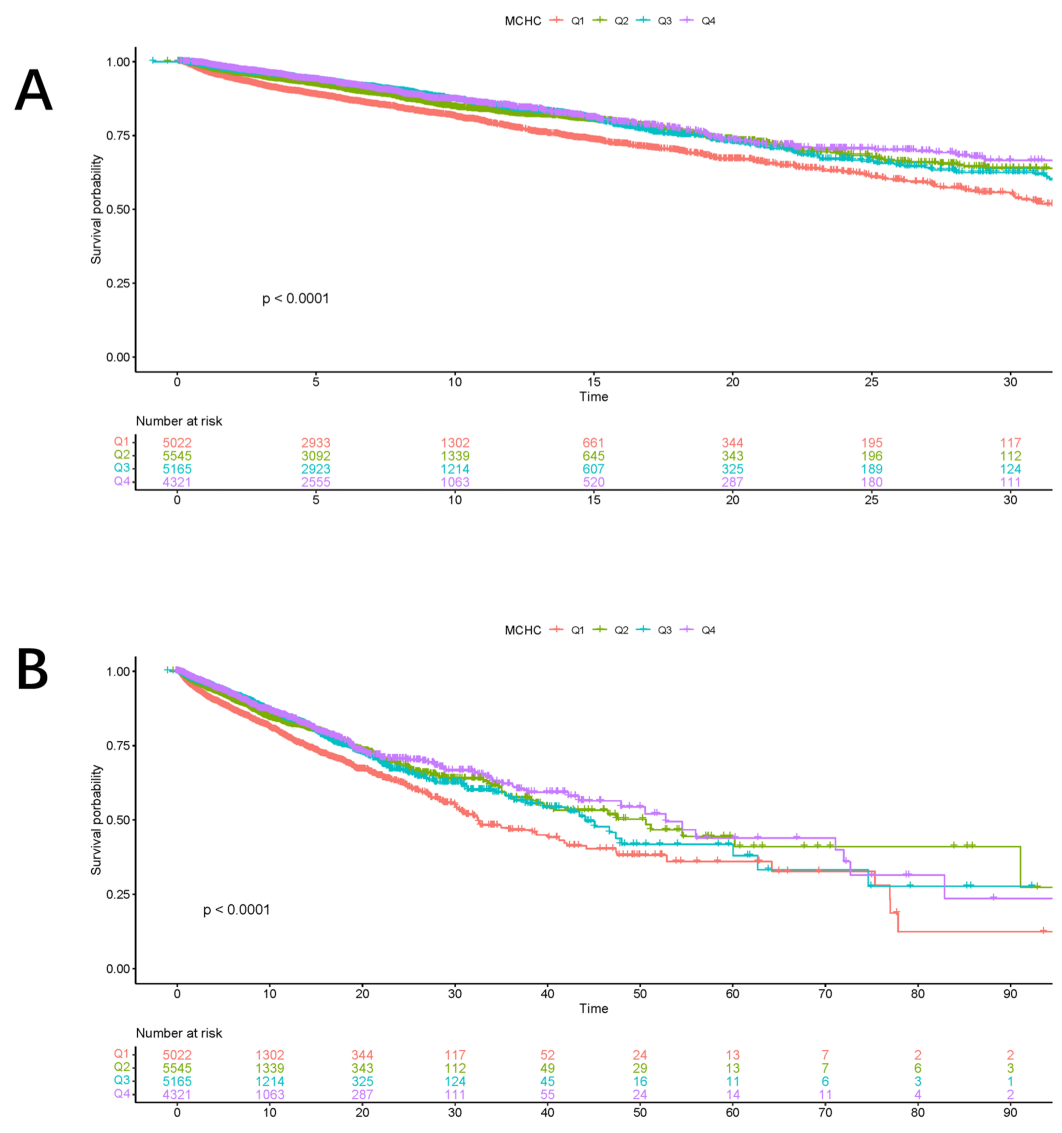
Cumulative mortality according to the Kaplan–Meier curves. (A) Thirty-day mortality. (B) Ninety-day mortality. MCHC: mean corpuscular hemoglobin concentration.

Kaplan-Meier survival analysis was used to assess outcomes over time (Figure 2). The resulting curves clearly visualize survival probabilities across the different patient groups stratified by MCHC quartile.

In the primary analysis, both 30-day (Figure 2A) and 90-day (Figure 2B) hospital mortality differed significantly among the four MCHC quartiles. This stratification allowed for a direct comparison of survival across MCHC gradations.

These results suggest that higher MCHC levels are associated with improved short-term survival. This association may reflect a more favorable physiological reserve or greater clinical stability at ICU admission.

Cox proportional hazard ratios for all-cause mortality

Cox proportional hazards analysis was conducted to evaluate the relationship between MCHC and in-hospital mortality (Table 2).

**Table 2:** Cox regression analysis of MCHC and mortality in cancer patients. Crude model: unadjusted. Model 1: Adjusted for sex, age, and weight. Model 2: Adjusted for sex, age, weight, CCI, SAPS II, and SOFA. Model 3: Adjusted for sex, age, weight, CCI, SAPS II, SOFA, sodium, glucose, serum creatinine, WBC, RBC, platelets, hemoglobin, sepsis, diabetes, arterial fibrillation, respiratory failure, heart failure, epinephrine, dopamine, and vasopressin. MCHC: mean corpuscular hemoglobin concentration; SOFA: Sequential Organ Failure Assessment; CCI: Charlson Comorbidity Index; SAPS II: Simplified Acute Physiology Score II; WBC: white blood cells; RBC: red blood cells.

Categories	Crude model		Model 1		Model 2		Model 3	
	95% CI	P	95% CI	P	95% CI	P	95% CI	P
Hospital mortality in 30 days								
Continuous variable per unit	0.89 (0.86, 0.91)	<0.0001	0.88 (0.86, 0.91)	<0.0001	0.89 (0.87, 0.92)	<0.0001	0.86 (0.83, 0.89)	<0.0001
Quartile								
Q1	Ref.		Ref.		Ref.		Ref.	
Q2	0.73 (0.66, 0.81)	<0.0001	0.72 (0.65, 0.79)	<0.0001	0.83 (0.75, 0.92)	<0.001	0.79 (0.71, 0.87)	<0.0001
Q3	0.65 (0.59, 0.73)	<0.0001	0.64 (0.58, 0.72)	<0.0001	0.74 (0.67, 0.83)	<0.0001	0.69 (0.62, 0.78)	<0.0001
Q4	0.63 (0.56, 0.70)	<0.0001	0.62 (0.55, 0.70)	<0.0001	0.64 (0.57, 0.72)	<0.0001	0.56 (0.49, 0.63)	<0.0001
p for trend								<0.001
Hospital mortality in 90 days								
Continuous variable per unit	0.89 (0.86, 0.91)	<0.0001	0.88 (0.86, 0.91)	<0.0001	0.89 (0.87, 0.92)	<0.0001	0.86 (0.83, 0.88)	<0.0001
Quartile								
Q1	Ref.		Ref.		Ref.		Ref.	
Q2	0.72 (0.66, 0.80)	<0.0001	0.71 (0.65, 0.79)	<0.0001	0.82 (0.74, 0.91)	<0.001	0.78 (0.70, 0.87)	<0.0001
Q3	0.66 (0.59, 0.73)	<0.0001	0.65 (0.58, 0.72)	<0.0001	0.75 (0.67, 0.83)	<0.0001	0.7 (0.62, 0.78)	<0.0001
Q4	0.63 (0.56, 0.70)	<0.0001	0.62 (0.56, 0.70)	<0.0001	0.64 (0.57, 0.71)	<0.0001	0.55 (0.49, 0.62)	<0.0001
p for trend								<0.001

To this end, we first verified the proportional hazards assumption through Schoenfeld residuals and found no evidence of violation for MCHC across follow-up intervals. After confirming model adequacy, we fitted a series of Cox models that progressively adjusted for potential confounders, thereby allowing us to isolate the independent effect of MCHC on the hazard of death.

MCHC, modeled as a continuous variable, was a consistent and independent predictor of 30-day mortality. The risk increased by 11% for every 1 g/dL decrease in MCHC in the crude model (hazard ratio (HR): 0.89, 95% CI: 0.86-0.91; p < 0.0001). Following full adjustment, the magnitude of this effect increased, with a 14% rise in hazard per 1 g/dL decrement (adjusted HR: 0.86, 95% CI: 0.83-0.89; p < 0.0001).

In the categorical analysis, the highest MCHC group showed a significantly elevated risk of in-hospital mortality compared to the lowest group. To create clinically interpretable contrasts, we divided MCHC into quartiles based on the distribution in the overall cohort and used the lowest quartile as the reference; in the unadjusted model, the HR was 0.63 (95% CI: 0.56-0.70; p < 0.0001). The graded pattern persisted after full covariate adjustment, with the middle quartile showing an intermediate hazard and the highest quartile retaining statistically significant protection against mortality; in the fully adjusted model, the HR was 0.56 (95% CI: 0.49-0.63; p < 0.0001).

Extending follow-up to 90 days did not attenuate the signal; rather, the effect size mirrored that observed for 30-day mortality, indicating that the prognostic value of MCHC is sustained over a longer horizon; in the unadjusted model, the HR was 0.89 (95% CI: 0.86-0.91, p < 0.0001). Multivariable adjustment again produced only a marginal change in point estimate while precision improved, underscoring the independence of MCHC from measured confounders; in the fully adjusted model, the HR was 0.86 (95% CI: 0.83-0.88, p < 0.0001).

Kaplan-Meier curves stratified by MCHC quartile showed early and sustained separation over the 90-day follow-up (log-rank p < 0.0001 for both crude and adjusted comparisons). The unadjusted HR was 0.63 (95% CI: 0.56-0.70; p < 0.0001). This survival advantage associated with higher MCHC remained consistent across follow-up durations and after multivariable adjustment, with a fully adjusted HR of 0.55 (95% CI: 0.49-0.62; p < 0.0001), reinforcing the durability and robustness of the association.

Subgroup analysis

Table 3 shows the stratified analysis of the association between MCHC and mortality, highlighting its predictive value for in-hospital mortality outcomes in different patient groups. To construct these strata, we prespecified clinically relevant subgroups based on primary admission diagnosis, baseline organ-function status, and demographic characteristics, then refitted the fully adjusted Cox model within each stratum while retaining the identical covariate set used in the main analysis; this approach ensures that observed effect modifications are not confounded by differential adjustment.

**Table 3:** HR for all-cause mortality in different subgroups. Note: "-" indicates empty cells. AF: atrial fibrillation; RF: respiratory failure; HF: heart failure; HR: hazard ratio; DM: diabetes mellitus.

Character	HR (95% CI)	p	p for interaction
Age			0.717
>65	0.872 (0.841, 0.904)	<0.0001	-
≤65	0.882 (0.838, 0.929)	<0.0001	-
Gender			<0.0001
No	0.933 (0.893, 0.974)	0.002	-
Yes	0.815 (0.783, 0.849)	<0.0001	-
HF			0.02
No	0.866 (0.840, 0.894)	<0.0001	-
Yes	0.974 (0.887, 1.071)	0.588	-
AF			0.034
No	0.863 (0.837, 0.891)	<0.0001	-
Yes	0.951 (0.875, 1.033)	0.232	-
RF			<0.001
No	0.876 (0.842, 0.912)	<0.0001	-
Yes	0.971 (0.929, 1.016)	0.205	-
Sepsis			0.156
No	0.868 (0.839, 0.899)	<0.0001	-
Yes	0.912 (0.860, 0.967)	0.002	-
Stroke			0.34
No	0.865 (0.840, 0.892)	<0.0001	-
Yes	0.927 (0.807, 1.066)	0.287	-
DM			0.949
No	0.872 (0.845, 0.899)	<0.0001	-
Yes	0.874 (0.797, 0.960)	0.005	-

The higher the level of MCHC, the lower the risk of death in cancer patients. Within the cancer cohort, defined as patients with an active malignancy documented at ICU admission, the dose-response relationship remained monotonic: compared with the lowest MCHC quartile, the middle quartile exhibited a 22% reduction in hazard, and the highest quartile achieved a 38% reduction, yielding a statistically significant trend test (p < 0.001) that corroborates the protective role of elevated MCHC even in the setting of neoplastic disease.

## Discussion

In this retrospective cohort study based on a large, multicenter critical care database, we systematically investigated the association between MCHC and mortality in ICU patients with cancer.

The Kaplan-Meier survival curves showed a clear separation among patients stratified by MCHC quartiles, with patients in the highest MCHC group exhibiting significantly better 30-day and 90-day survival compared with those in the lowest MCHC group. These findings suggest that MCHC measured at ICU admission may reflect important physiological differences that are closely related to prognosis in critically ill cancer patients. The consistent trend observed across both short-term and longer-term mortality endpoints further supports the stability of this association.

Previous studies have reported associations between MCHC and prognosis in various critical and non-critical conditions, including acute myocardial infarction, acute pulmonary embolism, hypertension, and hepatorenal syndrome. However, evidence regarding the prognostic significance of MCHC in critically ill patients with cancer has been limited [4-7]. The present study extends existing knowledge by demonstrating that MCHC is also significantly associated with mortality in this high-risk population, highlighting its potential role as a readily available and clinically useful prognostic indicator in the ICU setting.

The detrimental impact of anemia on patient prognosis extends beyond the context of lung cancer, significantly affecting outcomes in a range of other malignancies, including gastric and colorectal cancers. This relationship is further corroborated by investigational studies, which demonstrate that anemia, even in its mildest forms, serves as a reliable predictor of worse postoperative survival outcomes among patients undergoing treatment for gastric cancer [10]. Furthermore, in colorectal cancer, preoperative anemia is similarly linked to a spectrum of adverse clinical outcomes. Collectively, these studies from diverse oncological domains emphasize the critical importance of recognizing and actively managing anemia as a modifiable factor to improve overall cancer prognosis.

Anemia's prognostic significance is also evident in breast and cervical cancers. In breast cancer patients undergoing neoadjuvant chemotherapy, pretreatment anemia correlates with lower pathological complete response rates and poorer long-term survival outcomes [11]. Similarly, in cervical cancer, both pre-treatment and on-treatment anemia are significant predictors of worse progression-free survival (PFS) and overall survival (OS), with improvements in hemoglobin levels during treatment associated with better outcomes [12].

The MCHC is a hematological parameter that has garnered attention for its potential prognostic value in various cancers. Recent studies have explored the association between MCHC and cancer prognosis, revealing significant correlations that could inform clinical decision-making [13]. Similarly, the prognostic significance of MCHC has been observed in hepatocellular carcinoma (HCC). A study involving 289 HCC patients undergoing hepatectomy found that lower MCHC values were significantly associated with larger tumor diameters and vascular invasion, both of which are indicators of aggressive disease. The study concluded that low MCHC was linked to poorer prognostic outcomes, with an HR of 0.372 for OS and 0.450 for PFS, suggesting that MCHC could serve as a valuable preoperative prognostic marker in HCC [14].

From a clinical perspective, MCHC is a routinely measured and inexpensive laboratory parameter, making it easily accessible in daily practice. The findings of this study suggest that MCHC may provide additional prognostic information beyond traditional severity scores and laboratory indices. Incorporating MCHC into early risk stratification may help clinicians better identify cancer patients at higher risk of adverse outcomes during ICU hospitalization.

Limitations

Limitations of this retrospective study include its observational design, potential residual confounding, the use of a single MCHC measurement, and data sourced solely from US ICUs, which may affect generalizability. Nevertheless, the findings highlight MCHC as a practical prognostic marker worthy of prospective validation.

## Conclusions

Our study found that lower admission MCHC independently predicted increased 30-day and 90-day mortality in cancer patients admitted to the ICU. This association remained robust after comprehensive adjustment for demographics, comorbidities, illness severity scores, laboratory parameters, and vasoactive medication use, suggesting that MCHC captures prognostic information beyond that provided by conventional risk stratification tools. The consistency of this relationship across both short-term and intermediate-term mortality endpoints, as well as its stability across diverse clinical subgroups stratified by age, sex, cancer type, and severity of illness, underscores the reliability and generalizability of MCHC as a prognostic indicator in this vulnerable population.
